# Effect of Weight-Reduction in Obese Mice Lacking Toll-Like Receptor 5 and C57BL/6 Mice Fed a Low-Fat Diet

**DOI:** 10.1155/2015/852126

**Published:** 2015-11-23

**Authors:** Shao-Chun Wu, Cheng-Shyuan Rau, Tsu-Hsiang Lu, Siou-Ling Tzeng, Yi-Chan Wu, Chia-Jung Wu, Chia-Wei Lin, Ching-Hua Hsieh

**Affiliations:** ^1^Department of Anesthesiology, Kaohsiung Chang Gung Memorial Hospital and Chang Gung University College of Medicine, Taoyuan City, Taiwan; ^2^Department of Neurosurgery, Kaohsiung Chang Gung Memorial Hospital and Chang Gung University College of Medicine, Taoyuan City, Taiwan; ^3^Department of Plastic and Reconstructive Surgery, Kaohsiung Chang Gung Memorial Hospital and Chang Gung University College of Medicine, Taoyuan City, Taiwan

## Abstract

*Background*. This study aims to investigate the effect of feeding low-fat diet (LFD) to diet-induced obesity (DIO) mice lacking TLR5 (TLR5^−/−^), which have a tendency to develop glucose intolerance with increased adiposity, compared to that in C57BL/6 mice.* Results*. TLR5^−/−^ and C57BL/6 male mice were divided into three subgroups: (1) control, mice were fed a standard AIN-76A (fat: 11.5 kcal%) diet for 12 weeks; (2) DIO, mice were fed a 58 kcal% high-fat diet (HFD) for 12 weeks; and (3) diet, mice were fed a HFD for 8 weeks to induce obesity and then switched to a 10.5 kcal% LFD for 4 weeks. The glucose intolerance in DIO TLR5^−/−^ mice was more significant than that in DIO C57BL/6 mice and was not attenuated by a switch to the LFD. Weight-reduction with LFD had significantly decreased the epididymal fat mass in C57BL/6 mice but not in TLR5^−/−^ mice. In addition, the LFD-fed TLR5^−/−^ mice showed significantly higher expression of ghrelin in the serum and resistin in the epididymal fat than that in C57BL/6 mice.* Conclusions*. This study demonstrated that* TLR5* gene knockout impairs some effects of weight-reduction in DIO.

## 1. Introduction

Obesity is associated with insulin resistance and an abnormal inflammatory response [1, 2]. High-fat uptake leads to metabolic alterations in the adipose tissue that is associated with the synthesis and release of a huge amount of proinflammatory adipokines and cytokines such as leptin, resistin, PAI-1, IL-6, IL-10, and TNF-*α* [2]. Increased level of the circulating free fatty acids also leads to macrophage activation and the production of proinflammatory cytokines via Toll-like receptors (TLRs) [3]. The adipocytes and preadipocytes isolated from the adipose tissues of the* ob/ob* and* db/db* mice, which are leptin and leptin-receptor-deficient, respectively, were characterized by significant upregulation of* TLR1* to* TLR9* expression than that with wild type cells [4–6]. Upregulated expressions of* TLR1* to* TLR9* and* TLR11* to* TLR13* are also observed in adipose tissues of high-fat diet- (HFD-) induced obese mice or leptin-deficient obese mice [7]. The magnitudes of the obesity-induced upregulation of the* TLR1*,* TLR4*,* TLR5*,* TLR8*,* TLR9,* and* TLR12* genes in the visceral adipose tissue were even greater in the diet-induced obesity (DIO) mice than in the* ob/ob* mice [7]. These upregulated expressions of TLRs in the expanded adipose tissues of obese mice are linked with downstream NF-*κ*B, IRFs, and STAT-1 activation and upregulated expressions of cytokines and chemokines via MyD88-dependent and MyD88-independent cascades [7].

Evidence collected from these inbred mouse strains suggests that the detrimental effects in metabolism due to HFD are strain dependent, and some strains such as C57BL/6J and C57BL/6N are genetically predisposed to metabolic defects resulting from HFD feeding [8, 9]. When C57BL/6J mice were fed a high-fat diet (HFD) with* ad libitum*, they developed insulin resistance and obesity in a manner that resembles disease progression in humans [10]. Furthermore, mice lacking Toll-like receptor 5 (TLR5), on a mixed C57BL/6J and C57BL/6N genetic background, develop insulin resistance and increased adiposity [11–13]. These mice exhibit hyperphagia and develop hallmark features of metabolic syndrome including hyperlipidemia, hypertension, insulin resistance, and increased adiposity [13]. The TLR5^−/−^ mice consumed about 10% more food, had greater stool output, and did not significantly impact the efficiency of dietary energy harvest than wild type littermates [13]. The TLR5^−/−^ mice also exhibited a reduced response to exogenous insulin relative to wild type mice. In addition, the insulin resistance of TLR5^−/−^ mice is not entirely dependent on increased food consumption or adiposity seeing that the lean TLR5^−/−^ mice after 12 weeks of food restriction regimen still exhibited a decreased response to exogenous insulin [13]. TLR5 is a transmembrane protein that is highly expressed in the intestinal mucosa and recognizes bacterial flagellin. HFD and bacteria interact to promote early inflammatory changes in the small intestine that contribute to the development of susceptibility to obesity and insulin resistance [12]. The low-grade proinflammatory signaling in TLR5-deficient mice may attenuate insulin signaling, resulting in increased food consumption that drives other manifestations of metabolic syndrome [13]. Moreover, the humans with the segregation of a dominant nonsense polymorphism (R392X, rs5744168) lack TLR5 function and become susceptible to type 2 diabetes [14].

Decreased energy intake and increased energy expenditure, which reduces adiposity and restore insulin sensitivity, are the two most commonly recommended lifestyle changes to treat DIO and its related disorders [15]. Calorie restriction is effective in improving the insulin sensitivity and decreasing both body weight and percent body fat [16]. Moreover, switching from a HFD to a low-fat diet (LFD) can reduce body weight and improve insulin sensitivity by reducing the percentage of fat in a diet [17]. Although food restriction prevents obesity, but not insulin resistance, in TLR5-deficient mice [13], limited information is known regarding the LFD effect on TLR5-deficient obese mice. In this study, we aim to investigate the diet effect of LFD feeding on the DIO mice lacking TLR5 against C57BL/6 mice.

## 2. Materials and Methods

### 2.1. Animal Experiments

C57BL/6NCrl mice were purchased from BioLasco (Taipei, Taiwan). TLR5^−/−^ (B6.129S1-Tlr5tm1Flv/J) mice were purchased from Jackson Laboratory (Bar Harbor, ME, USA). Animals were housed, and surgical procedures, including analgesia, were performed in Association for Assessment and Accreditation of Laboratory Animal Care International-accredited SPF facility according to national and institutional guidelines to minimize the suffering of affected animals. Animal protocols were approved by the IACUC of Chang Gung Memorial Hospital, Taiwan (permission number 2012091002). In this experiment, 24 male TLR5^−/−^ and 24 male C57BL/6NCrl mice were randomly assigned to three subgroups (*n* = 8 in each group) as follows: (1) control, where mice were fed a standard AIN-76A (fat: 11.5 kcal%) diet with* ad libitum* for 12 weeks; (2) DIO, where mice were fed a 58 kcal% HFD (D12331; Research Diets Inc., New Brunswick, NJ) with* ad libitum* for 12 weeks to induce obesity; and (3) diet, where mice were fed a 58 kcal% HFD (D12331)* ad libitum* for 8 weeks to induce obesity and then fed a 10.5 kcal% LFD (D 12329; Research Diets Inc.) for 4 weeks. Weight measurements were recorded weekly, and an intraperitoneal glucose tolerance test (IPGTT) was performed at the beginning and end of the experiment to confirm that HFD-fed mice developed an obese and insulin-resistant phenotype. Briefly, mice were allowed to fast for 5 h, and baseline blood glucose levels were measured with an Accu-Check Advantage blood glucose meter (Roche, NJ) using blood samples collected from the tail vein. Mice were injected intraperitoneally with 2 g of glucose (in sterile PBS) per kilogram body weight. The glucose level was measured via tail vein blood (~10 *μ*L) at *t* = −30 and 0 (pre) and *t* = 15, 30, 60, 90, and 120 min after the glucose infusion. Data were averaged and graphed as blood glucose level as a function of time. To reflect the circulating levels of glucose during the glucose tolerance test, we calculated the total area under the curve (AUC) of the glucose concentration versus time by the linear trapezoidal rule for the period of 0–120 min after glucose infusion. In the end of the experiment, all mice were euthanized, and the epididymal white adipose tissue (WAT) of each mouse was collected and weighed. The adipose tissue block embedded in paraffin was sectioned at 5 *μ*m to measure the adipocyte area. Three 5 *μ*m-thick sections of the same fat specimen at 50 *μ*m distance were mounted on glass plate and stained with hematoxylin and eosin. Two different microscopic fields (magnification ×100) per plate were photographed and 100 adipose cells were arbitrarily selected in the center of field and their cell diameters were assessed by tracing the outline of each adipocyte. The mean adipocyte area was measured from the epididymal WAT of control and experimental mice (*n* = 8 in each group) using Image-Pro Plus image analysis software (Carl Zeiss, Oberkochen, Germany) and expressed in terms of square micrometers. The liver embedded in paraffin was sectioned at 5 *μ*m and stained with hematoxylin and eosin. At the end of the experiment, 1 mL of whole blood was collected via cardiac puncture into a plain tube and allowed to clot for 1 h. Samples were centrifuged at 3000 ×g for 10 min, and sera were aliquoted and stored at −80°C until further analysis.

### 2.2. Cytokine Assays

Cytokine concentrations in the serum and epididymal WAT were analyzed using Bio-Plex Cytokine Assay (Mouse Diabetes 8-Plex, cat. number 171-F7001M, BioRad, Hercules, CA) including ghrelin, gastric inhibitory polypeptide (GIP), glucagon-like peptide-1 (GLP-1), insulin, leptin, plasminogen activator inhibitor type 1 (PAI-1), glucagon, and resistin, which are potentially involved in obesity-associated diabetes. Expressions of IL-6, IL-10, and TNF-*α* in the adipose tissue were also assessed using the Bio-Plex system (BioRad). Assays were performed on four biological replicates as per the manufacturer's instructions. Results were expressed in picograms per milliliter of serum or per milligram of adipose tissue.

### 2.3. Statistical Analysis

All experimental data are expressed as the mean ± standard error of the mean. Analysis of variance combined with a Bonferroni post hoc correction was performed to identify significant differences in body weight, weight of fat, adipocyte area, glucose levels, and serum cytokine levels. *p* ≥ 0.05 was regarded as the level of statistical significance.

## 3. Results

### 3.1. LFD Reduces the Body Weight Gain by HFD Feeding

**Figure 1:**
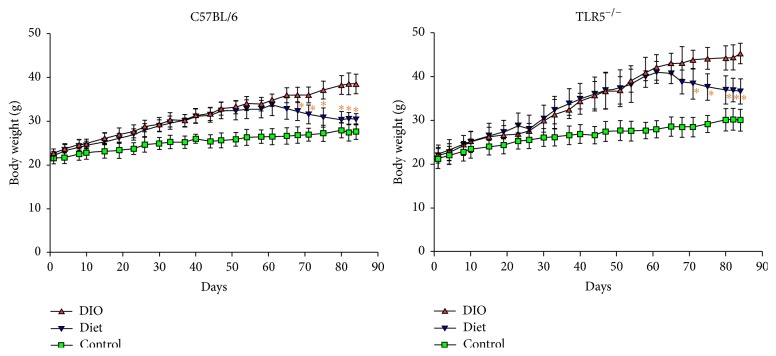
The body weight of C57BL/6 and TLR5^−/−^ mice on a weekly basis till week 12 (*n* = 8). Control: the mice fed with the standard diet; DIO: the mice fed with high-fat diet; diet: the mice fed with high-fat diet for 8 weeks to induce obesity and then we change to low-fat diet for subsequent 4 weeks. ^*∗*^  (red color), *p* < 0.05 versus DIO.

HFD-fed C57BL/6 and TLR5^−/−^ mice gained more body weight compared to regular chow, as shown in Figure 1. At the end of the experiment, the difference in body weight gained between the DIO and control groups reached 10.8 g in C57BL/6 mice (38.5 ± 1.2 versus 27.7 ± 1.8 g) and 15.3 g in TLR5^−/−^ mice (45.3 ± 2.3 versus 30.0 ± 2.5 g). The TLR5^−/−^ mice gained more weight by HFD feeding than C57BL/6 mice at week 12. The LFD-induced weight-reduction in DIO mice became significant at week 2 and week 3 for C57BL/6 and TLR5^−/−^ mice, respectively, and the body weight decreased by 8.1 g in C57BL/6 mice (38.5 ± 1.2 versus 30.4 ± 1.3 g) and by 8.7 g in TLR5^−/−^ mice (45.3 ± 2.3 versus 36.6 ± 2.9 g) 4 weeks later. After 4 weeks of LFD feeding, 21.0% and 19.2% reduction in body weight of the DIO C57BL/6 and TLR5^−/−^ mice, respectively, were recorded.

### 3.2. TLR5^−/−^ Present Severe Glucose Intolerance Than C57BL/6 Mice

**Figure 2:**
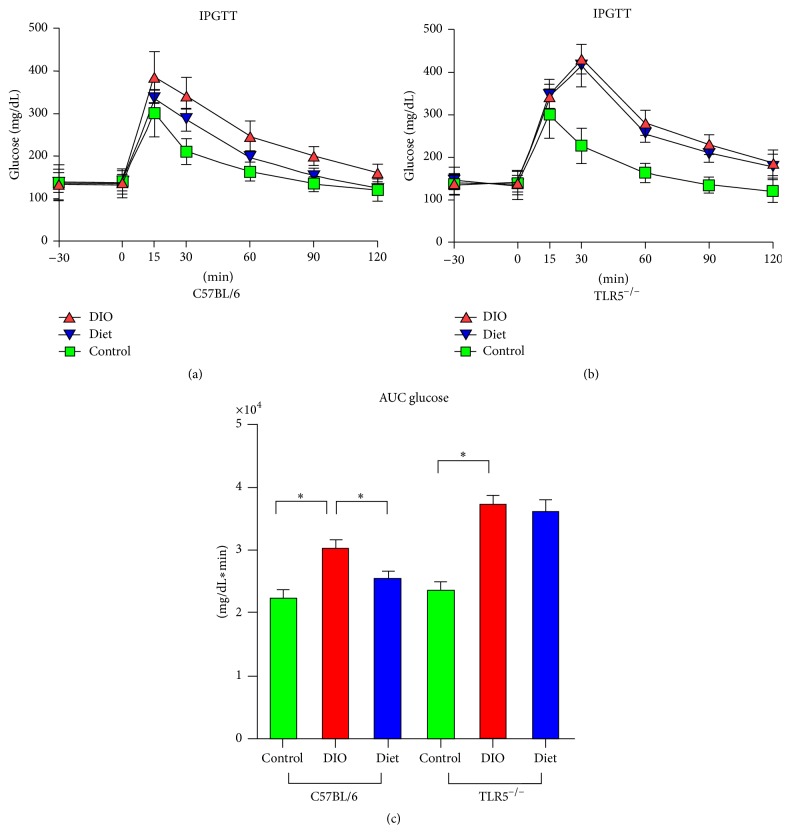
Intraperitoneal blood glucose concentrations of (a) C57BL/6 and (b) TLR5^−/−^ mice and (c) the quantification of area under the curve (AUC) during a 120 min intraperitoneal glucose tolerance test (IPGTT) in groups of control, DIO, and diet at week 12. ^*∗*^ *p* < 0.05.

In the control C57BL/6 mice, glucose infusion increased the blood glucose levels to a peak of 300 mg/dL after 15 min and then gradually returned to baseline after 120 min (Figure 2). HFD-fed animals displayed significantly higher blood glucose concentrations at 30 to 120 min during the IPGTT compared to control mice, which is evident by a 36% higher incremental glucose AUC. In addition, significantly lower glucose level was observed in LFD-fed mice at 30 and 60 min after the glucose infusion against the HFD-fed mice, resulting in around 15% lower glucose AUC. In control TLR5^−/−^ mice, the response to glucose infusion was quite similar to that in C57BL/6 mice. In DIO TLR5^−/−^ mice, glucose infusion increased blood glucose levels to a peak of 420 mg/dL after 30 min and then gradually decreased but did not return to the baseline after 120 min. With a 60% higher incremental glucose AUC, the glucose intolerance in DIO TLR5^−/−^ mice was more significant compared to DIO C57BL/6 mice. Furthermore, unlike C57BL/6 mice, no significantly lower glucose level or glucose AUC was observed in LFD-fed TLR5^−/−^ mice during IPGTT after glucose infusion against DIO TLR5^−/−^ mice.

### 3.3. TLR5^−/−^ Present Different Adiposity Response to Weight-Reduction Than C57BL/6 Mice

**Figure 3:**
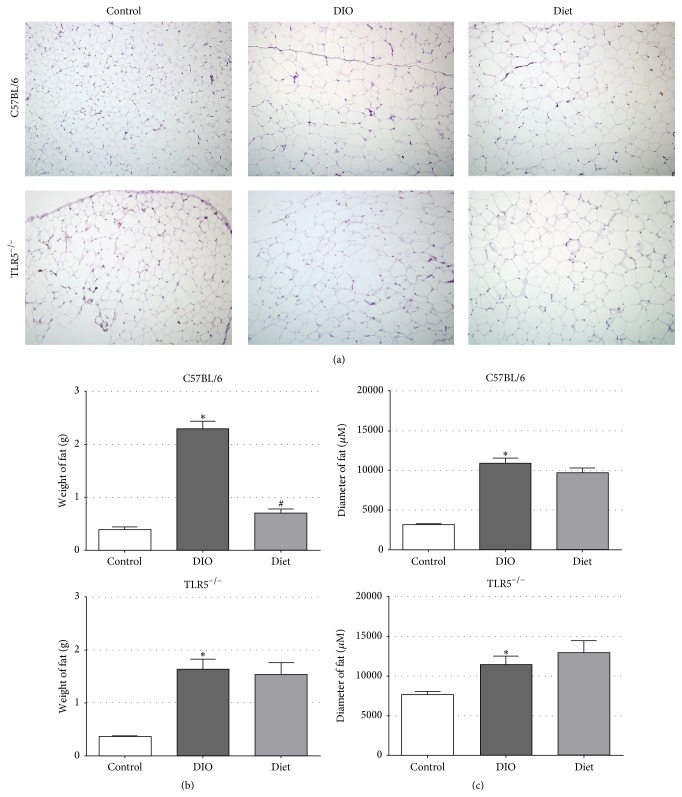
(a) Hematoxylin and eosin stain of the paraffin-embedded epididymal white adipose tissue at 5 *μ*m section of C57BL/6 and TLR5^−/−^ mice in groups of control, DIO, and diet at week 12. (b) The weight of epididymal white adipose tissue and (c) adipocyte area of C57BL/6 and TLR5^−/−^ mice in groups of control, DIO, and diet at week 12. ^*∗*^ *p* < 0.05 versus control. ^#^ *p* < 0.05 versus DIO.

As revealed in Figure 3, the histologic examination of epididymal fat demonstrates that HFD increased the size of adipocytes and induced a significant level of adipocyte hypertrophy at the end of the experiment in both C57BL/6 and TLR5^−/−^ mice. HFD for 8 weeks increased the average epididymal fat mass by ~1.9 g and ~1.7 g in C57BL/6 and TLR5^−/−^ mice, respectively, compared to those fed on regular chow. Notably, histologic examination also revealed that the size of adipocytes was significantly larger in the control TLR5^−/−^ mice compared to C57BL/6 mice. In addition, although a switch to the LFD had significantly decreased the epididymal WAT in C57BL/6 mice, the decrease of epididymal fat mass was not significant in TLR5^−/−^ mice. Moreover, there was no reduction in the diameter of fat lobules after a switch to LFD feeding in both C57BL/6 and TLR5^−/−^ mice.

### 3.4. HFD Induces Severer Hepatic Steatosis in TLR5^−/−^ Than C57BL/6 Mice

**Figure 4:**
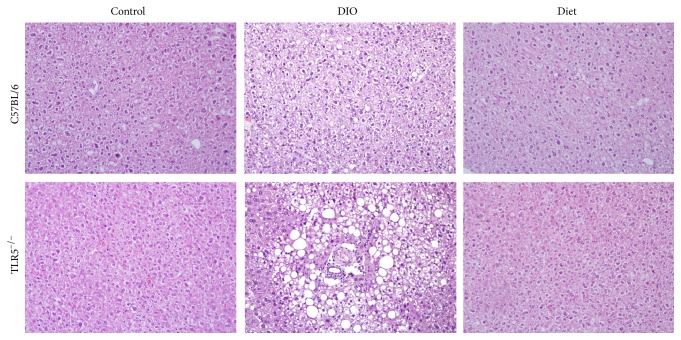
Hepatic steatosis in the hematoxylin and eosin stain of the paraffin-embedded liver at 5 *μ*m section of C57BL/6 and TLR5^−/−^ mice in groups of control, DIO, diet at week 12.

Hepatic steatosis is a common metabolic complication associated with obesity. In this study, the histological examination revealed that HFD induced progressively enlarged vacuoles, suggesting hepatic fat deposition in the liver of C57BL/6 and TLR5^−/−^ mice. Moreover, the liver of DIO TLR5^−/−^ mice had more hepatic fat deposition with various size of fat cells compared to DIO C57BL/6 mice (Figure 4). However, no fat deposition was found in the liver of C57BL/6 and TLR5^−/−^ mice after LFD feeding for 4 weeks.

### 3.5. TLR5^−/−^ Present Different Cytokine Response to Weight-Reduction Than C57BL/6 Mice in Serum

**Figure 5:**
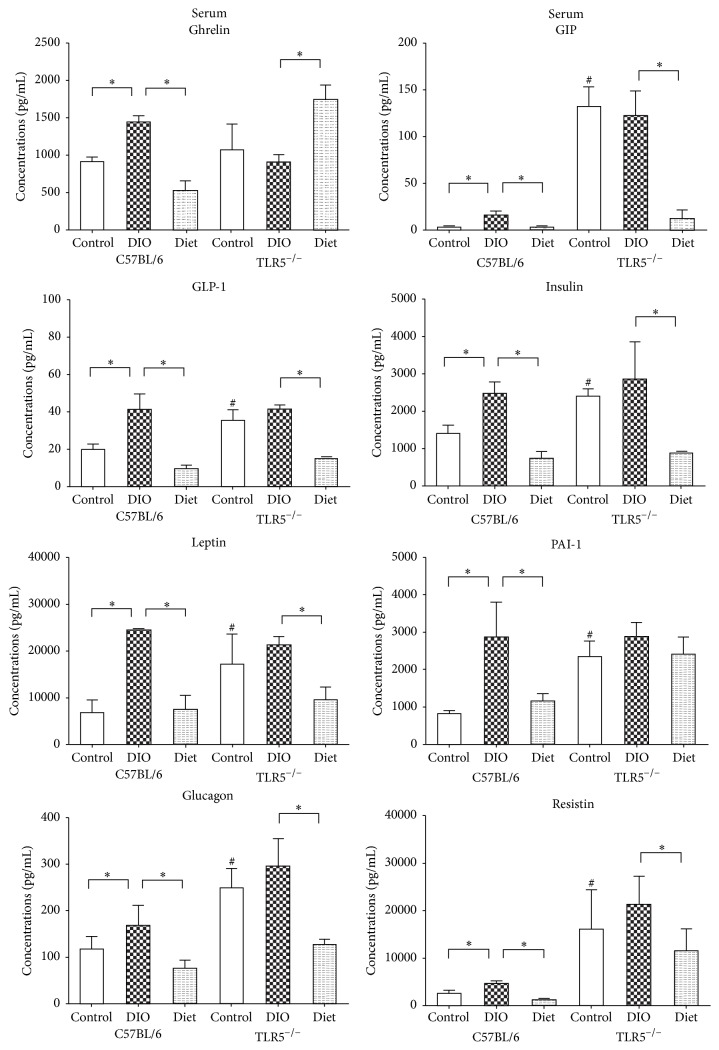
Concentrations of serum cytokines including ghrelin, gastric inhibitory polypeptide (GIP), glucagon-like peptide-1 (GLP-1), insulin, leptin, plasminogen activator inhibitor type 1 (PAI-1), glucagon, and resistin analyzed by the Bio-Plex Multiplex cytokine assay at week 12 in the C57BL/6 and TLR5^−/−^ mice in groups of control, DIO, and diet. ^*∗*^ *p* < 0.05 versus indicated group. ^#^ *p* < 0.05 versus control C57BL/6 mice.

DIO increased the expression of ghrelin, GIP, GLP-1, insulin, leptin, PAI-1, glucagon, and resistin (Figure 5) in the serum of C57BL/6 mice, and weight-reduction with LFD feeding significantly reduced their expressions. However, in TLR5^−/−^ mice, GIP, GLP-1, insulin, leptin, PAI-1, glucagon, and resistin were already higher in the control mice compared to C57BL/6 control mice. DIO in the TLR5^−/−^ mice was not associated with the upregulation of the cytokines ghrelin, GIP, GLP-1, insulin, leptin, PAI-1, glucagon, and resistin. Weight-reduction via LFD feeding significantly reduced the expression of circulating GIP, GLP-1, insulin, leptin, glucagon, and resistin in the TLR5^−/−^ mice but not ghrelin and PAI-1. The LFD-fed TLR5^−/−^ mice showed significantly higher ghrelin expression in the serum compared to DIO TLR5^−/−^ mice.

### 3.6. TLR5^−/−^ Present Different Cytokine Response to Weight-Reduction Than C57BL/6 Mice in Fat

**Figure 6:**
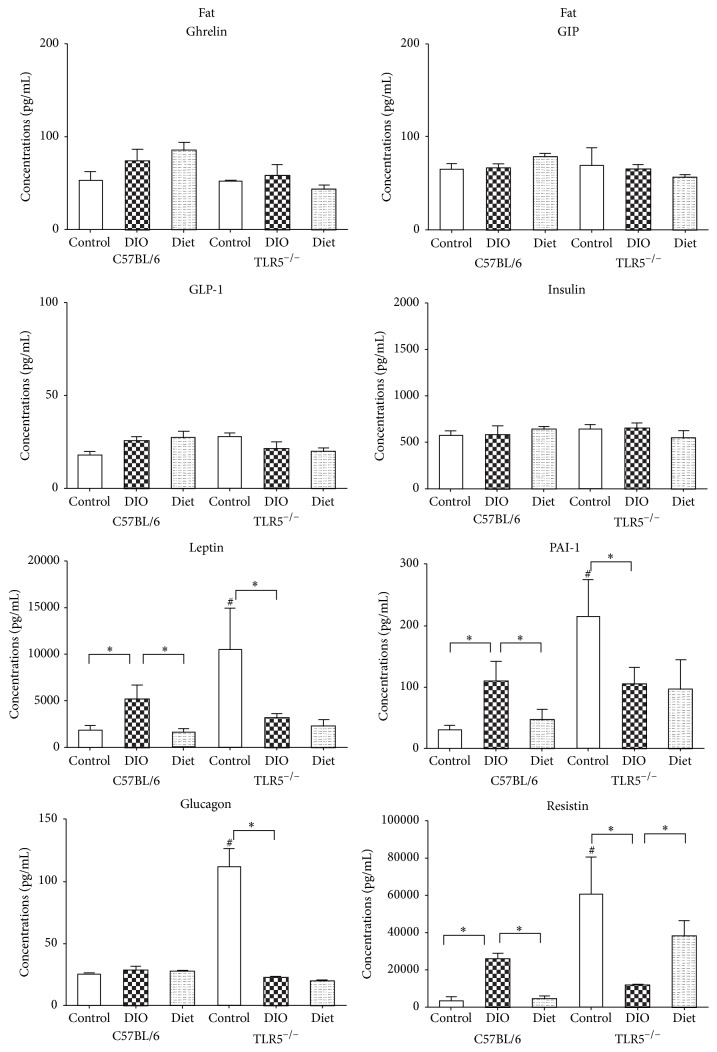
Concentrations of adipose tissue cytokines analyzed by the Bio-Plex Multiplex cytokine assay at week 12 in C57BL/6 and TLR5^−/−^ mice in groups of control, DIO, and diet. ^*∗*^ *p* < 0.05 versus indicated group. ^#^ *p* < 0.05 versus control C57BL/6 mice.

**Figure 7:**
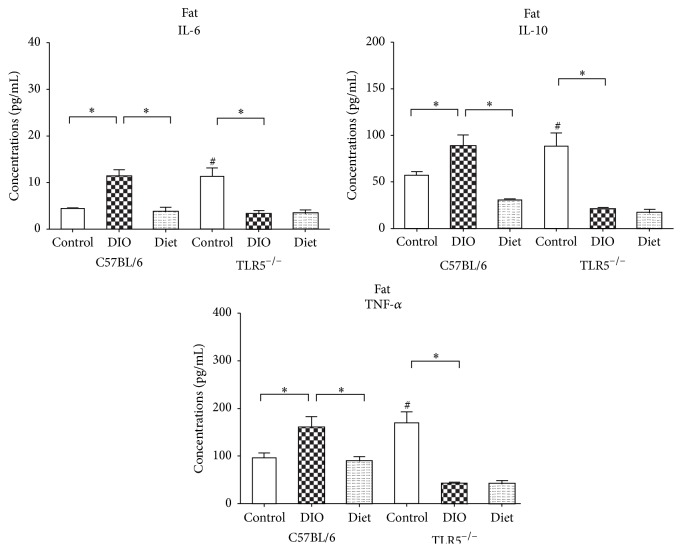
Concentrations of adipose tissue cytokines including IL-6, IL-10, and TNF-*α* at week 12 in C57BL/6 and TLR5^−/−^ mice in groups of control, DIO, and diet. ^*∗*^ *p* < 0.05 versus indicated group. ^#^ *p* < 0.05 versus control C57BL/6 mice.

In C57BL/6 mice, DIO increased the expression of leptin, PAI-1, and resistin (Figure 6) as well as IL-6, IL-10, and TNF-*α* (Figure 7) in the epididymal WAT. Weight-reduction via LFD feeding significantly reduced their expression. In TLR5^−/−^ mice, leptin, PAI-1, glucagon, and resistin as well as IL-6, IL-10, and TNF-*α* were already higher in the control mice than those in the C57BL/6 control mice. In contrast, DIO in the TLR5^−/−^ mice results in significant downregulation of leptin, PAI-1, glucagon, resistin, IL-6, IL-10, and TNF-*α*. In addition, weight-reduction via LFD feeding resulted in an increased expression of resistin in the epididymal WAT of the TLR5^−/−^ mice, but no significant change in the expression of leptin, PAI-1, glucagon, IL-6, IL-10, and TNF-*α* was found.

## 4. Discussion

In this study, HFD feeding significantly increased the body weight and adipocyte size in both TLR5^−/−^ and C57BL/6 mice. After 12 weeks of HFD feeding, the TLR5^−/−^ mice gained more weight and showed significantly higher glucose intolerance and hepatic steatosis than C57BL/6 mice. Although, switching to a LFD is effective in weight-reduction and improves metabolic health parameters in obesity [18], weight-reduction with LFD in the TLR5^−/−^ mice resulted in a different response regarding the change of weight of epididymal WAT, glucose tolerance, and cytokines response in the serum and adipose tissue compared to the C57BL/6 mice.

Visceral fat is a highly active tissue from the metabolic point of view [19, 20]. Nowadays it is assumed that unfavorable changes in the secretion of adipose tissue hormones and inflammatory cytokines caused by obesity influence the development of metabolic syndrome [21]. Excess of visceral adipose tissue and increased production of adipokines are mostly responsible for metabolic complications [21]. As the big adipocytes are more prone to rupture and therefore obviously constitute a focus of inflammation, a positive correlation between adipocyte size and TNF-*α*, IL-6, and C-reactive protein was reported [22]. Adipose inflammation and ectopic fat deposition in extra-adipose tissues collectively resulted in impaired glucose homeostasis [23] and several lines of evidence prove that chronic inflammation causatively contributes to insulin resistance development in obesity [24, 25]. Additionally, because approximately 75% of weight lost by dieting is composed of adipose tissue [26], it is believed that the health benefits that result from weight loss are due to the reductions in proinflammatory secretions by adipocytes and the influence those secretions have on cell types in other tissues [27–29]. In this study, although a switch to the LFD had significantly decreased the epididymal fat mass in C57BL/6 mice, this effect was not found in TLR5^−/−^ mice. Moreover, glucose intolerance was still significant in LFD-fed TLR5^−/−^ mice than C57BL/6 mice. However, there was no reduction in the diameter of fat lobules after a switch to LFD feeding both in C57BL/6 and in TLR5^−/−^ mice.

In this study, the LFD-induced weight-reduction resulted in a different cytokine response in serum and adipose tissues of TLR5^−/−^ and C57BL/6 mice. In C57BL/6 mice, weight-reduction via LFD feeding could significantly reduce the DIO-related expression of ghrelin, GIP, GLP-1, insulin, leptin, PAI-1, glucagon, and resistin in the serum and PAI-1, resistin, IL-6, IL-10, and TNF-*α* in the epididymal WAT. In the TLR5^−/−^ mice, although LFD feeding significantly reduced the expression of circulating GIP, GLP-1, insulin, leptin, glucagon, and resistin, no such effect was found in the ghrelin and PAI-1. The LFD-fed TLR5^−/−^ mice showed significantly higher ghrelin expression in the serum compared to the DIO mice. In addition, weight-reduction via LFD feeding resulted in an increased expression of resistin in the epididymal WAT of the TLR5^−/−^ mice but there were no significant changes in the expression of leptin, PAI-1, glucagon, IL-6, IL-10, and TNF-*α*.

Among these investigated cytokines, the difference in response of ghrelin, PAI-1, and resistin between TLR5^−/−^ and C57BL/6 mice during the weight-reduction gained much attention. Ghrelin and the ghrelin receptor are expressed by lymphocytes, monocytes, and dendritic cells; therefore, there was no significant change in ghrelin expression in the fat deposits of TLR5^−/−^ or C57BL/6 mice during DIO with HFD and weight-reduction with LFD. Potent anti-inflammatory effects of ghrelin were reported on the expression of IL-1*β*, IL-6, and TNF*α* in the liver, spleen, lungs, and mesenteric lymph nodes of LPS-treated mice associated with an attenuation of the LPS-induced anorexia [30]. Activation of the ghrelin receptor also results in an inhibition of proinflammatory cytokine expression and an increase in survival in various inflammatory disease models [31, 32]. The strong correlation of plasma level of PAI-1 with body mass index (BMI) and visceral accumulation of body fat suggests that PAI-1 is an adipose tissue-derived circulating peptide [33]. A direct correlation between the expression of PAI-1 in adipocytes and its serum concentration has been observed [34–36]. In addition, increased concentration of PAI-1 was found in the blood of obese patients, with some exhibiting insulin resistance [37]. Concentration of PAI-1 in blood decreases with weight-reduction via increased physical activity and caloric restriction [38]. The reduction in PAI-1 levels after weight loss is more associated with the degree of weight loss than with triglyceride or insulin changes [39]. Resistin is predominantly expressed in adipocyte and immunocompetent cells as a proinflammatory cytokine and participate in obesity-associated inflammation [40–42]. Increased resistin concentration has been observed in mice with genetically and diet-induced obesity [43]. In obese individuals, the amount of resistin in adipose tissue, especially visceral adipose tissue, is significantly higher compared to individuals with normal weight [43, 44]. It has also been reported that increased serum resistin levels in obese patients with insulin resistance [45] and resistin impair glucose homeostasis and insulin action in mice [44, 46].

The variation in the expression of cytokines during weight-reduction with LFD feeding is yet to be explored. It may be attributed to the significantly higher expression of cytokines in the serum (GIP, GLP-1, insulin, leptin, PAI-1, glucagon, and resistin) and in the fat (leptin, PAI-1, glucagon, resistin, IL-6, IL-10, and TNF-*α*) deposits of the control TLR5^−/−^ mice. Because the proteins measured in this study might originate from myriad cell types, including adipocytes, immune and epithelial cells, it is hard to ascertain the source of production. However, there was no evidence that TLR5 plays the role of a direct upstream mediator of the differently expressed ghrelin, PAI-1, and resistin. In this study, whether there are different expressions of IL-1*β*, IL-18, and IL-22, which had been reported to be involved in metabolic disorder and insulin sensitivity [47–49], is interesting but yet investigated. Notably, it had been reported that the mice deficient in IL-22 receptor are prone to developing metabolic disorders after the feeding with HFD [49]. In addition, the administration of exogenous IL-22 in genetically obese leptin-receptor-deficient (*db/db*) mice and mice fed with HFD reverses many of the metabolic symptoms, including hyperglycemia and insulin resistance [49]. However, whether there is similar effect of IL-22 administration in the TLR5^−/−^ mice, which had already a higher expression of leptin that differs from the* db/db* mice, warrants further investigation.

Gastrointestinal tract plays an important role in DIO and other nutrition-related disorders, as it represents the route by which all nutrients and other sources of energy are ingested, processed, and absorbed [50]. Microbiota play an important role in the complex network of molecular and cellular interactions that link genotype to phenotype and have potential implications for obesity and diabetes. Evidence of the connection between overall gut microbial composition and obesity had been provided [51, 52]. In addition, emerging literature has implicated HFD-induced alterations in gut microbiota in the obesity epidemic [53]. Notably, development of obesity in genetically or diet-induced obese mice is associated with dramatic changes in the composition and metabolic function of the microbiota. This trait is transmissible as colonization of germ-free mice with an “obese-gut-derived” microflora results in a much greater increase in total body fat and leads to obesity [11, 12]. In addition, gut microbiota of TLR5^−/−^ and wild type littermate mice were significantly different in their species composition [13]. Transplantation of TLR5^−/−^ microbiota into wild type germ-free mice conferred many aspects of the TLR5^−/−^ phenotype to the wild type germ-free hosts, including hyperphagia, obesity, hyperglycemia, insulin resistance, and elevated levels of proinflammatory cytokines [13]. Lean TLR5^−/−^ mice exhibited a decreased response to exogenous insulin, which suggests that their insulin resistance is not entirely dependent on increased food consumption or adiposity [13]. Therefore, the study of the change and its impact on the gut microbiota of TLR5^−/−^ mice during weight-reduction with LFD feeding might provide more important information regarding the distinct effects of weight-reduction in obese TLR5^−/−^ and C57BL/6 mice.

## 5. Conclusion

In conclusion, this study demonstrates that weight-reduction with LFD resulted in a difference in response in TLR5^−/−^ and C57BL/6 mice regarding the change of epididymal fat weight, glucose tolerance, and cytokines response in the serum and adipose tissue. These results also indicate that the knockout of* TLR5* gene impaired some effect of weight-reduction in DIO.

## References

[1] Tsukumo D. M. L., Carvalho-Filho M. A., Carvalheira J. B. C. (2007). Loss-of-function mutation in toll-like receptor 4 prevents diet-induced obesity and insulin resistance. *Diabetes*.

[2] Monteiro R., Azevedo I. (2010). Chronic inflammation in obesity and the metabolic syndrome. *Mediators of Inflammation*.

[3] Betanzos-Cabrera G., Estrada-Luna D., Belefant-Miller H., Cancino-Diaz J. C. (2012). Mice fed with a high fat diet show a decrease in the expression of ‘toll like receptor (TLR)2 and TLR6 mRNAs in adipose and hepatic tissues. *Nutricion Hospitalaria*.

[4] Schäffler A., Schölmerich J., Salzberger B. (2007). Adipose tissue as an immunological organ: Toll-like receptors, C1q/TNFs and CTRPs. *Trends in Immunology*.

[5] Pietsch J., Batra A., Stroh T. (2006). Toll-like receptor expression and response to specific stimulation in adipocytes and preadipocytes: on the role of fat in inflammation. *Annals of the New York Academy of Sciences*.

[6] Batra A., Pietsch J., Fedke I. (2007). Leptin-dependent toll-like receptor expression and responsiveness in preadipocytes and adipocytes. *The American Journal of Pathology*.

[7] Kim S.-J., Choi Y., Choi Y.-H., Park T. (2012). Obesity activates toll-like receptor-mediated proinflammatory signaling cascades in the adipose tissue of mice. *Journal of Nutritional Biochemistry*.

[8] Podrini C., Cambridge E. L., Lelliott C. J. (2013). High-fat feeding rapidly induces obesity and lipid derangements in C57BL/6N mice. *Mammalian Genome*.

[9] Collins S., Martin T. L., Surwit R. S., Robidoux J. (2004). Genetic vulnerability to diet-induced obesity in the C57BL/6J mouse: physiological and molecular characteristics. *Physiology and Behavior*.

[10] Surwit R. S., Kuhn C. M., Cochrane C., McCubbin J. A., Feinglos M. N. (1988). Diet-induced type II diabetes in C57BL/6J mice. *Diabetes*.

[11] Tilg H. (2010). Obesity, metabolic syndrome and microbiota: multiple interactions. *Journal of Clinical Gastroenterology*.

[12] Ding S., Lund P. K. (2011). Role of intestinal inflammation as an early event in obesity and insulin resistance. *Current Opinion in Clinical Nutrition and Metabolic Care*.

[13] Vijay-Kumar M., Aitken J. D., Carvalho F. A. (2010). Metabolic syndrome and altered gut microbiota in mice lacking toll-like receptor 5. *Science*.

[14] Al-Daghri N. M., Clerici M., Al-Attas O. (2013). A nonsense polymorphism (R392X) in TLR5 protects from obesity but predisposes to diabetes. *Journal of Immunology*.

[15] Sartipy P., Loskutoff D. J. (2003). Monocyte chemoattractant protein 1 in obesity and insulin resistance. *Proceedings of the National Academy of Sciences of the United States of America*.

[16] Weiss E. P., Racette S. B., Villareal D. T. (2006). Improvements in glucose tolerance and insulin action induced by increasing energy expenditure or decreasing energy intake: a randomized controlled trial. *The American Journal of Clinical Nutrition*.

[17] Muurling M., Jong M. C., Mensink R. P. (2002). A low-fat diet has a higher potential than energy restriction to improve high-fat diet-induced insulin resistance in mice. *Metabolism: Clinical and Experimental*.

[18] Hoevenaars F. P. M., Keijer J., Herreman L. (2014). Adipose tissue metabolism and inflammation are differently affected by weight loss in obese mice due to either a high-fat diet restriction or change to a low-fat diet. *Genes & Nutrition*.

[19] Bertin E., Nguyen P., Guenounou M., Durlach V., Potron G., Leutenegger M. (2000). Plasma levels of tumor necrosis factor-alpha (TNF-*α*) are essentially dependent on visceral fat amount in type 2 diabetic patients. *Diabetes & Metabolism*.

[20] You T., Nicklas B. J., Ding J. (2008). The metabolic syndrome is associated with circulating adipokines in older adults across a wide range of adiposity. *Journals of Gerontology A Biological Sciences and Medical Sciences*.

[21] Gnacińska M., Małgorzewicz S., Stojek M., Łysiak-Szydłowska W., Sworczak K. (2009). Role of adipokines in complications related to obesity: a review. *Advances in Medical Sciences*.

[22] Monteiro R., de Castro P. M. S. T., Calhau C., Azevedo I. (2006). Adipocyte size and liability to cell death. *Obesity Surgery*.

[23] Gao M., Ma Y., Liu D., Stadler K. (2015). High-fat diet-induced adiposity, adipose inflammation, hepatic steatosis and hyperinsulinemia in outbred CD-1 mice. *PLoS ONE*.

[24] Wu Y., Wu T., Wu J. (2013). Chronic inflammation exacerbates glucose metabolism disorders in C57BL/6J mice fed with high-fat diet. *Journal of Endocrinology*.

[25] Olefsky J. M., Glass C. K. (2009). Macrophages, inflammation, and insulin resistance. *Annual Review of Physiology*.

[26] Ballor D. L., Poehlman E. T. (1994). Exercise-training enhances fat-free mass preservation during diet-induced weight loss: a meta-analytical finding. *International Journal of Obesity*.

[27] González O., Tobia C., Ebersole J. L., Novak M. J. (2012). Caloric restriction and chronic inflammatory diseases. *Oral Diseases*.

[28] Redman L. M., Ravussin E. (2011). Caloric restriction in humans: impact on physiological, psychological, and behavioral outcomes. *Antioxidants & Redox Signaling*.

[29] Ye J., Keller J. N. (2010). Regulation of energy metabolism by inflammation: a feedback response in obesity and calorie restriction. *Aging*.

[30] Dixit V. D., Schaffer E. M., Pyle R. S. (2004). Ghrelin inhibits leptin- and activation-induced proinflammatory cytokine expression by human monocytes and T cells. *The Journal of Clinical Investigation*.

[31] Taub D. D. (2008). Neuroendocrine interactions in the immune system. *Cellular Immunology*.

[32] Taub D. D. (2007). Novel connections between the neuroendocrine and immune systems: the ghrelin immunoregulatory network. *Vitamins and Hormones*.

[33] Alessi M. C., Peiretti F., Morange P., Henry M., Nalbone G., Juhan-Vague I. (1997). Production of plasminogen activator inhibitor 1 by human adipose tissue: possible link between visceral fat accumulation and vascular disease. *Diabetes*.

[34] Mertens I., Van Gaal L. F. (2002). Obesity, haemostasis and the fibrinolytic system. *Obesity Reviews*.

[35] Michalska M., Iwan-Zietek I., Gnilka W. (2013). PAI-1 and *α*2-AP in patients with morbid obesity. *Advances in Clinical and Experimental Medicine*.

[36] Schneider D. J., Sobel B. E. (2012). PAI-1 and diabetes: a journey from the bench to the bedside. *Diabetes Care*.

[37] Vague P., Juhan-Vague I., Aillaud M. F. (1986). Correlation between blood fibrinolytic activity, plasminogen activator inhibitor level, plasma insulin level, and relative body weight in normal and obese subjects. *Metabolism*.

[38] Belalcazar L. M., Ballantyne C. M., Lang W. (2011). Metabolic factors, adipose tissue, and plasminogen activator inhibitor-1 levels in type 2 diabetes: findings from the look AHEAD study. *Arteriosclerosis, Thrombosis, and Vascular Biology*.

[39] Folsom A. R., Qamhieh H. T., Wing R. R. (1993). Impact of weight loss on plasminogen activator inhibitor (PAI-1), factor VII, and other hemostatic factors in moderately overweight adults. *Arteriosclerosis, Thrombosis, and Vascular Biology*.

[40] Bo S., Gambino R., Pagani A. (2005). Relationships between human serum resistin, inflammatory markers and insulin resistance. *International Journal of Obesity*.

[41] Reilly M. P., Lehrke M., Wolfe M. L., Rohatgi A., Lazar M. A., Rader D. J. (2005). Resistin is an inflammatory marker of atherosclerosis in humans. *Circulation*.

[42] Bokarewa M., Nagaev I., Dahlberg L., Smith U., Tarkowski A. (2005). Resistin, an adipokine with potent proinflammatory properties. *The Journal of Immunology*.

[43] McTernan P. G., Kusminski C. M., Kumar S. (2006). Resistin. *Current Opinion in Lipidology*.

[44] Steppan C. M., Lazar M. A. (2004). The current biology of resistin. *Journal of Internal Medicine*.

[45] Baranova A., Gowder S. J., Schlauch K. (2006). Gene expression of leptin, resistin, and adiponectin in the white adipose tissue of obese patients with non-alcoholic fatty liver disease and insulin resistance. *Obesity Surgery*.

[46] Nakata M., Okada T., Ozawa K., Yada T. (2007). Resistin induces insulin resistance in pancreatic islets to impair glucose-induced insulin release. *Biochemical and Biophysical Research Communications*.

[47] Netea M. G., Joosten L. A. B., Lewis E. (2006). Deficiency of interleukin-18 in mice leads to hyperphagia, obesity and insulin resistance. *Nature Medicine*.

[48] Stienstra R., Joosten L. A. B., Koenen T. (2010). The inflammasome-mediated caspase-1 activation controls adipocyte differentiation and insulin sensitivity. *Cell Metabolism*.

[49] Wang X., Ota N., Manzanillo P. (2014). Interleukin-22 alleviates metabolic disorders and restores mucosal immunity in diabetes. *Nature*.

[50] Wolfe M. M., Boylan M. O. (2014). Obesity and the gastrointestinal tract: you are what you eat. *Journal of Clinical Gastroenterology*.

[51] Ley R. E., Turnbaugh P. J., Klein S., Gordon J. I. (2006). Microbial ecology: human gut microbes associated with obesity. *Nature*.

[52] Turnbaugh P. J., Ley R. E., Mahowald M. A., Magrini V., Mardis E. R., Gordon J. I. (2006). An obesity-associated gut microbiome with increased capacity for energy harvest. *Nature*.

[53] Murphy E. A., Velazquez K. T., Herbert K. M. (2015). Influence of high-fat diet on gut microbiota: a driving force for chronic disease risk. *Current Opinion in Clinical Nutrition and Metabolic Care*.

